# The relationship between duration of access to intravenous oxycodone self-administration, economic demand for oxycodone, and cognition in rats

**DOI:** 10.21203/rs.3.rs-9349824/v1

**Published:** 2026-04-23

**Authors:** Sydney Dick, Amy Heaton, Aaron D. Claypool, Katherine E. Driver, Elizabeth A. Colley, Viktoria Galbava, Marek Schwendt, Lori A. Knackstedt

**Affiliations:** University of Florida; University of Florida; University of Florida; University of Florida; University of Florida; University of Florida; University of Florida; University of Florida

**Keywords:** addiction, cognition, substance use disorder, cortex, economic demand, relapse

## Abstract

**Rationale:**

Individuals who use opioids display cognitive deficits.

**Objectives:**

Here we used oxycodone intravenous self-administration (IVSA) to test the hypothesis that cognitive deficits would be observed in rats following opioid exposure and would worsen with increased oxycodone-seeking.

**Methods:**

Male and female Sprague-Dawley rats were trained to self-administer oxycodone or food. Rats either underwent short-access (ShA; 3 hr/day) or long-access (LgA; 6 hr/day) oxycodone IVSA. Control rats underwent 3 hr/day food self-administration. Next, rats underwent economic demand analysis wherein the number of responses required to obtain a reinforcer was increased. After 14 days of abstinence, rats were tested for cue-primed relapse of reinforcer-seeking. Rats then underwent novel object recognition (NOR) testing followed by assessment of cognitive flexibility using the probabilistic reversal learning (PRL) task. Rats were perfused following the last PRL session for assessment of cortical activity via c-fos immunohistochemistry.

**Results:**

While LgA rats had greater oxycodone intake, there were no effects of access length on demand elasticity or cued seeking. Modest effects of oxycodone exposure were found on cognition, including increased NOR performance in LgA rats and different PRL strategies. Despite equivalent behavior shown during the final PRL session, ShA rats displayed increased c-fos expression in the orbitofrontal cortex (OFC).

**Conclusions:**

While oxycodone self-administration does not overtly impair PRL performance, more activity in the OFC is necessary to maintain the same level of performance. Furthermore, length of daily oxycodone access is not a determinant of economic demand for oxycodone.

## Introduction

Persistent problematic use of opioid drugs remains a significant public health concern worldwide. Cognition has consistently been found to be impaired by opioid use across several cognitive domains comprising executive function (Ornstein et al. 2000; Ersche et al. 2005b; Fishbein et al. 2007; Baldacchino et al. 2012; Kroll et al. 2018; Baldacchino et al. 2019). Impaired cognition has been linked to an increased propensity to seek drugs and is thus a clinically relevant deleterious consequence (Fox et al. 2009; Vonmoos et al. 2013; Verdejo-Garcia et al. 2014).

One of several cognitive domains found to be impaired in persons who use opioids is that of cognitive flexibility (Hekmat et al. 2010; Ahn et al. 2014; Myers et al. 2016; Regier et al. 2024). Deficits in cognitive flexibility have been observed in persons who use heroin and those on opioid replacement therapies (opioid agonists such as methadone or buprenorphine) relative to healthy controls. Even in the absence of overt deficits in opioid groups, differences in decision-making strategies have been found (Ahn et al. 2014; Myers et al. 2016). A second domain that is impaired in the context of opioid use disorder (OUD) is that of recognition memory (Ornstein et al. 2000; Ersche et al. 2006; Fishbein et al. 2007; Kroll et al. 2018; Tolomeo et al. 2019; Regier et al. 2024).

Animal models are essential for the identification of neural circuits and pharmacological targets of psychiatric disease, including opioid use disorder and the cognitive impairments that accompany it. While previous research has found detrimental effects of opioid administration on cognitive flexibility (Anderson et al. 2021) and recognition memory (Zhong et al. 2018; Wan et al. 2023) in rodents, none have investigated how individual differences in the opioid taking and seeking impact cognitive flexibility. Here we used operant intravenous self-administration (IVSA) of oxycodone followed by the novel object recognition (NOR) task and an operant probabilistic reversal learning (PRL) task to assess object recognition and cognitive flexibility, respectively, to test the hypothesis that greater oxycodone intake and seeking would be associated with increased deficits in reversal learning and recognition memory. To manipulate the amount of oxycodone intake, rats were provided either short access (ShA) or long access (LgA) to oxycodone IVSA daily prior to assessment of economic demand for oxycodone and cued seeking after 2 weeks of abstinence.

We also tested the hypothesis that any observed deficits in reversal learning would be accompanied by changes in cortical activity. Performance on the PRL task relies on a corticostriatal network that supports flexible updating of behavior when reinforcement contingencies are changed. In rodents, the orbitofrontal cortex (OFC), the prelimbic (PL) prefrontal cortex (PFC), and the anterior cingulate cortex (Cg1) are involved in aspects of PRL. Disrupting OFC function impairs the updating of stimulus–reward associations, or the reversal of a learned rule (Boulougouris et al. 2007; Dalton et al. 2016; Amodeo et al. 2017; Izquierdo et al. 2017). There is also evidence of functional specialization within the OFC, with lateral OFC contributing more strongly to reversal of stimulus-reward rules and feedback sensitivity, whereas medial OFC supports integration of prior information during probabilistic discrimination (Dalton et al. 2016; Izquierdo et al. 2017; Verharen et al. 2020). The PL influences win-stay/lose-shift strategies, and the Cg1 supports monitoring of outcomes and behavioral adjustment (Boulougouris et al. 2007; Izquierdo et al. 2017). Importantly, this circuitry appears conserved across species, including humans (Cools et al. 2002; Rudebeck and Murray 2008; Tsuchida et al. 2010). Dopaminergic signaling within corticostriatal circuits is necessary for reversal of behavioral strategies made to earn a reinforcer (Cools et al. 2001; Schirru et al. 2022). Together, findings across rodents, non-human primates, and humans converge on a circuitry involving the orbitofrontal and prefrontal cortices, modulated by limbic structures and dopaminergic signaling, that supports probabilistic reversal learning and behavioral flexibility. A history of drug intake alters dopamine signaling in this circuity (Volkow and Fowler 2000; Volkow et al. 2009), which may account for observed deficits in reversal learning. Thus, we assessed cortical activity following completion of the PRL task with the immediate early gene c-fos. c-Fos is widely used as a marker of recent neuronal activation because it is rapidly and transiently expressed following strong synaptic stimulation (Kovács 1998, 2008).

## Methods

### Animals

Male and female Sprague-Dawley rats were individually housed on a 12-hour reverse light cycle within a temperature-monitored vivarium. All procedures were conducted during the dark phase. Rats were divided into 3 groups upon arrival that underwent the following conditions: short-access oxycodone IVSA (“ShA”; n = 16); long-access oxycodone IVSA (“LgA”; n = 16), and food self-administration (“Control”; n = 14). Half of the rats in each condition were female. Rats were food restricted to 20 g/day. These experiments were approved by the University of Florida IACUC.

### Drugs

Oxycodone HCl (Sigma Aldrich, St. Louis, MO) was prepared in 0.9% physiological saline. Heparin (100 IU/mL; 0.2 mL) was used to flush catheters prior to and after each IVSA session to maintain catheter patency. The analgesic carprofen (5 mg/kg SC) and antibiotic cefazolin (100 mg/kg IV) were administered post-operatively. Catheter patency was assessed weekly using methohexital sodium (10 mg/mL IV).

### Surgery

All rats underwent implantation of intra-jugular catheters while anesthetized with ketamine (87.5 mg/kg) and xylazine (5 mg/kg). Catheters (Silastic, Dow Corning, MI) were inserted into the jugular vein, fastened with sutures and then extended subcutaneously between the shoulder blades where it was attached to a cannula (Plastics One, Roanoke, VA) connected to a rubber harness (Instech, Plymouth Meeting, PA).

### Self-administration, Economic Demand Analyses, & Cue-primed Relapse

**Figure 1:**
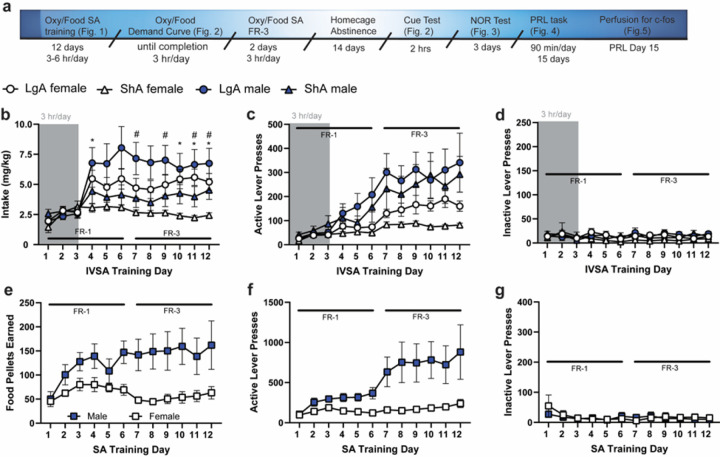
a. Timeline. b. There was a significant Sex × Access Length × Day interaction for the amount of oxycodone intake during IVSA, with both sexes increasing intake in the LgA condition. d. There were no differences in the number of active lever presses between ShA and LgA conditions, but males exhibited greater active lever pressing than females overall. d. Inactive lever presses did not differ between conditions. e. Male rats earned more food pellets than the female rats. f. Active lever presses for food pellets were greater in male rats. g. Inactive lever presses were not different between the sexes. * = p<0.05 Female LgA vs. Female ShA. # = p<0.05 Male LgA vs. Female ShA. All panels depict mean ± SEM.

Self-administration occurred in standard 2-lever operant chambers (Med Associates, Inc.). Delivery of a reinforcer occurred upon presses on the active lever and was accompanied by cues: a 2900 Hz tone and illumination of a stimulus light over the active lever. Reinforcer delivery was followed by a 20 sec time-out. No consequences were programmed for presses on the inactive lever. ShA and LgA rats began oxycodone intravenous self-administration (IVSA; 0.1 mg/kg/infusion) on a fixed-ratio (FR) – 1 schedule of reinforcement for 3 hr/day for 3 days. ShA rats continued 3 hr/day sessions for the subsequent 9 days, while LgA rats underwent 6 hr/day sessions for 9 days. Rats in both conditions underwent 6 days on an FR-1 schedule followed by 6 days on an FR-3 schedule. Control rats received food pellets as reinforcers (45 mg; Bioserv) on an FR-1 schedule for 3 hr/day for 6 days then an FR-3 schedule for 6 days. Following 12 days of training, economic demand for oxycodone and food was evaluated in daily 3 hr sessions. The “price” of the reinforcer (the FR required to receive one reinforcer) was increased by quarter log units (e.g., FR-10, FR-18, FR-32) every second day until zero reinforcers were delivered on two consecutive days of the same FR schedule. Rats then re-established SA for 2 days on an FR-3 schedule until the number of reinforcers earned was within 25% of the self-administration training average. Rats then underwent 14 days of abstinence before a 2 hr cue-test during which active lever presses only yielded cues associated with the reinforcer. See timeline in Fig. 1a.

### Novel object recognition (NOR) test

The NOR was conducted after the cue test (see Fig. 1a). A 41.4 cm × 41.4 cm square container made of black epoxide resin (Lavex Products, Clark Core Services, LLC; Lancaster, PA, USA) was used for NOR. The testing room was illuminated to ~ 58 lux with diffuse white light. NOR trials were recorded using a Logitech C290 Pro HD webcam. On Day 1, rats were habituated to the testing room for 20 minutes. On Day 2, rats were habituated to the empty arena for 5 minutes. On Day 3, rats underwent a 5-minute “Sample” trial, wherein rats were placed into the arena facing away from two identical objects (either object “A” or “B” placed 30.5 cm apart and affixed to the floor; see Fig. 3a). On Day 4, 24 hr after the Sample trial, rats experienced a “Retention” trial, wherein one familiar object (the same object presented during the Sample trial) and one novel object was presented. In order to counterbalance the presentation, 23 (n = 12 males, n = 11 females) rats were presented with object A as the familiar object for the Sample trial, and 23 rats (n = 11 males, n = 12 females) were presented with object B as the familiar object. The novel object was placed on either the right or left side in an alternating fashion. All rats were tested for NOR, however, data from 10 rats (1 male LgA; 3 female and 5 male ShA; 1 female and 1 male Control) could not be analyzed due to escaping the arena or moving the objects on Day 3. EthoVision^®^ XT 18 software was used to quantify the amount of time spent with each object.

### Probabilistic Reversal Learning (PRL)

Testing in the PRL task began 24 hr after NOR testing (see Fig. 1a). The PRL task was conducted in operant chambers that were located in a different room than those used for self-administration and utilized nose ports instead of levers. At the start of each session, one of the ports was arbitrarily designated the high-probability port, giving an 80% chance of reward and a 20% chance of a time-out while the second port gave a 20% chance of reward and 80% chance of a time-out and was considered the low-probability port. Illumination of the two nose poke ports and house light signaled the start of the task. When rats performed a “win” response, the lights of the nose poke ports were turned off, a 45 mg sucrose pellet (BioServ) was delivered, a 0.5 sec tone was generated, and the cue lights above the ports were illuminated. A new trial began once infrared light sensors detected the consumption of the pellet, and the cue lights were turned off. A “lose” response resulted in a 10 sec time-out signaled by the nose port lights and house lights being turned off. The choice made in the previous trial had no influence on the odds of reward for the following trial. In a “reversal trial”, the high-probability port became the low-probability port, and vice versa. A successful reversal was coded when 8 consecutive responses were made in the high-probability port. A higher number of reversals in one session is considered to be indicative of greater cognitive flexibility. The number of trials a rat could perform was unlimited. Sessions were 90 min in duration and were conducted over 15 days.

### Perfusion and Brain Preservation

Thirty minutes after the final 90-min PRL session, rats were transcardially perfused with 4% paraformaldehyde (PFA). This timing was chosen to assess the peak c-fos expression during the PRL task, as c-fos mRNA peaks at ~ 30 min and protein levels peak ~ 90 min post-stimuli (Kovács 1998, 2008). Once brains were extracted, they were preserved in 4% PFA for 24 hr prior to being transferred to a solution of 25% sucrose for the next 48 hr. Brains were then flash frozen with isopentane and stored at − 80°C and later coronally sliced (30 μM) using a cryostat. See below for regions of interest. Slices were stored in 0.01% sodium azide solution.

### Immunohistochemistry and Imaging

Free-floating brain sections containing the cortical regions of interest were blocked in 2% normal donkey serum before being incubated overnight in rabbit anti-c-fos antibody (1:10,000, Cell Signaling; cat. #2250S) at room temperature. The next day, slices were incubated in a biotinylated donkey anti-rabbit secondary antibody (1:500) for 2 hr, followed by avidin-biotin complex (Vector Laboratories), and visualization with 3,3-diaminobenzidine (Vector Laboratories). Sections were mounted onto slides and coverslipped. Images were obtained at 20x using an AmScope MU1803 CMOS camera mounted to an Olympus BX51 microscope. Two images/hemisphere from each region were acquired and c-fos expression quantified using NIH ImageJ software with a cell counter plug-in. Regions of interest included the PL, OFC, and Cg1 cortices based on their established roles in PRL as discussed above. Prior work examining OFC subregions in this task divided the OFC into the medial OFC (MO) and lateral OFC (LO) (Verharen et al. 2020). Here, we further divided the LO into three regions as shown in Fig. 5a – the anterior lateral OFC (aLO), posterior lateral OFC (pLO) and ventral OFC (VO), due to different, and sometimes opposing, roles for these regions (Cunningham and Redish 2025). The primary motor cortex (M1) was selected as a control region that we hypothesized would be involved in the number of nose pokes emitted but not cognition.

### Statistical Analysis

Dependent measures were compared between groups using ANOVAs, with Day considered a repeated measure (RM). Economic demand was assessed by plotting reinforcer deliveries as a function of FR and fitting the data with the equation: logQ = log(Q_0_) + k(e-αP – 1). Q is the number of reinforcer deliveries at each FR requirement, or “price (P)”. K is a fixed scaling parameter. Q_0_ estimates reinforcer consumption at an FR of 0 (i.e., when the drug is “free”). Alpha (α) is an index of elasticity of demand and is the rate of change in consumption as a function of FR. Larger α values reflect more elastic demand, such that consumption decreases more quickly with price increases, independent of consumption at null cost (Q_0_).

Essential Value [1/(100 × α × *k*^1.5^)] is the strength of the reinforcer’s demand. Group-level analyses compared demand elasticity between oxycodone access conditions and sex using a least sum of squares F test. Alpha, P_max_, Essential Value, and Q_0_ were calculated for each rat and compared between groups with Access Length × Sex ANOVAs for oxycodone rats and t-tests (male vs. female) for Control rats. For the NOR, the total amount of time spent with the nose within 2 cm of the object was quantified and compared between groups and tests. A discrimination ratio was calculated (time spent with novel object/time spent with both objects); positive values represent greater preference for the novel object. Because the ShA group only had 3 males successfully complete the NOR task, Sex was not used as factor in the NOR analyses. PRL dependent measures included the number of trials, reversals, rewards earned, win-stay and lose-shift choices; these were compared between conditions with Group × Time RM ANOVAs. Two multiple linear regressions were conducted to determine whether oxycodone taking and seeking measures (total oxycodone intake, active presses during relapse test, log alpha, P_max_ and Essential Value) were significant predictors of the variance in: 1) the discrimination index and 2) the total number of reversals/100 trials in all 15 days of the PRL task. c-fos expression/mm was averaged between images from the same region within each rat and then compared with 2-way Sex × Condition ANOVAs. For all analyses, alpha = 0.05. Tukey’s post-hoc tests were used to follow up on significant interactions. Outliers were defined as values more than 2 standard deviations from the mean and were eliminated from further analysis. Outlier values were only detected for c-fos expression.

## Results

### Self-administration

When comparing oxycodone intake (mg/kg) during the 9 days of differential access to oxycodone (“LgA” and “ShA”), there was a Sex × Access × Day interaction [F_(8, 224)_ = 2.571, p = 0.0106]. Post-hoc tests found that on several days, the female LgA group had greater intake than the female ShA group (Fig. 1b). While the LgA male rats had increased intake relative to the ShA females on several days (Fig. 1b), LgA males did not have greater intake than the ShA males on any day. A 2-way Sex × Access ANOVA computed on the total amount of oxycodone intake (mg/kg) during the 12-day training period found a significant effect of Access [F_(1, 29)_ = 8.938, p = 0.0056] but not Sex and no interaction; LgA rats displayed greater total intake than ShA rats. There was no Sex × Access × Day interaction for active lever presses across the 9 days, but there was a Sex × Day interaction [F_(8, 224)_ = 3.460, p = 0.0009], with males showing increased active lever pressing relative to females (Fig. 1c). Inactive lever presses were low and did not differ by sex or access condition (Fig. 1d). Male Controls earned more food pellets than the female Controls [Sex × Day interaction: F_(2.152, 25.83)_ = 3.363, p < 0.05; Fig. 1e]. Post-hoc tests did not find further group differences. Males exhibited greater active lever presses for food pellets [main effect of Sex; F_(1, 12)_ = 6.047, p = 0.031; Fig. 1f]. Inactive lever presses were not different between the sexes (Fig. 1g).

Due to an increase in the number of trials completed by LgA rats during the PRL task (see below), the number of active lever presses during self-administration was compared between Control, ShA and LgA groups within each sex separately. For males, a Group × Day ANOVA found no significant interaction but main effects of Group [F_(2, 20)_ = 4.528, p = 0.0239] and Day [F_(1.359, 27.18)_ = 14.58, p = 0.0002], with Control rats showing increased active lever presses relative to both oxycodone groups (Suppl. Fig. S1a). There were no group differences in inactive lever presses for males (Suppl. Fig. S1b). There was a significant Group × Day interaction [F_(6.077, 60.77)_ = 2.896, p = 0.047] for active lever presses in females, with Control rats showing increased presses relative to both oxycodone groups in the first several days of self-administration, and LgA rats increasing to be no different than Controls in later days (Suppl. Fig. S1d). There were no main effects of Group or Day for inactive lever presses for females (Suppl. Fig. S1d).

### Oxycodone and food seeking during economic demand and cued relapse conditions

**Figure 2:**
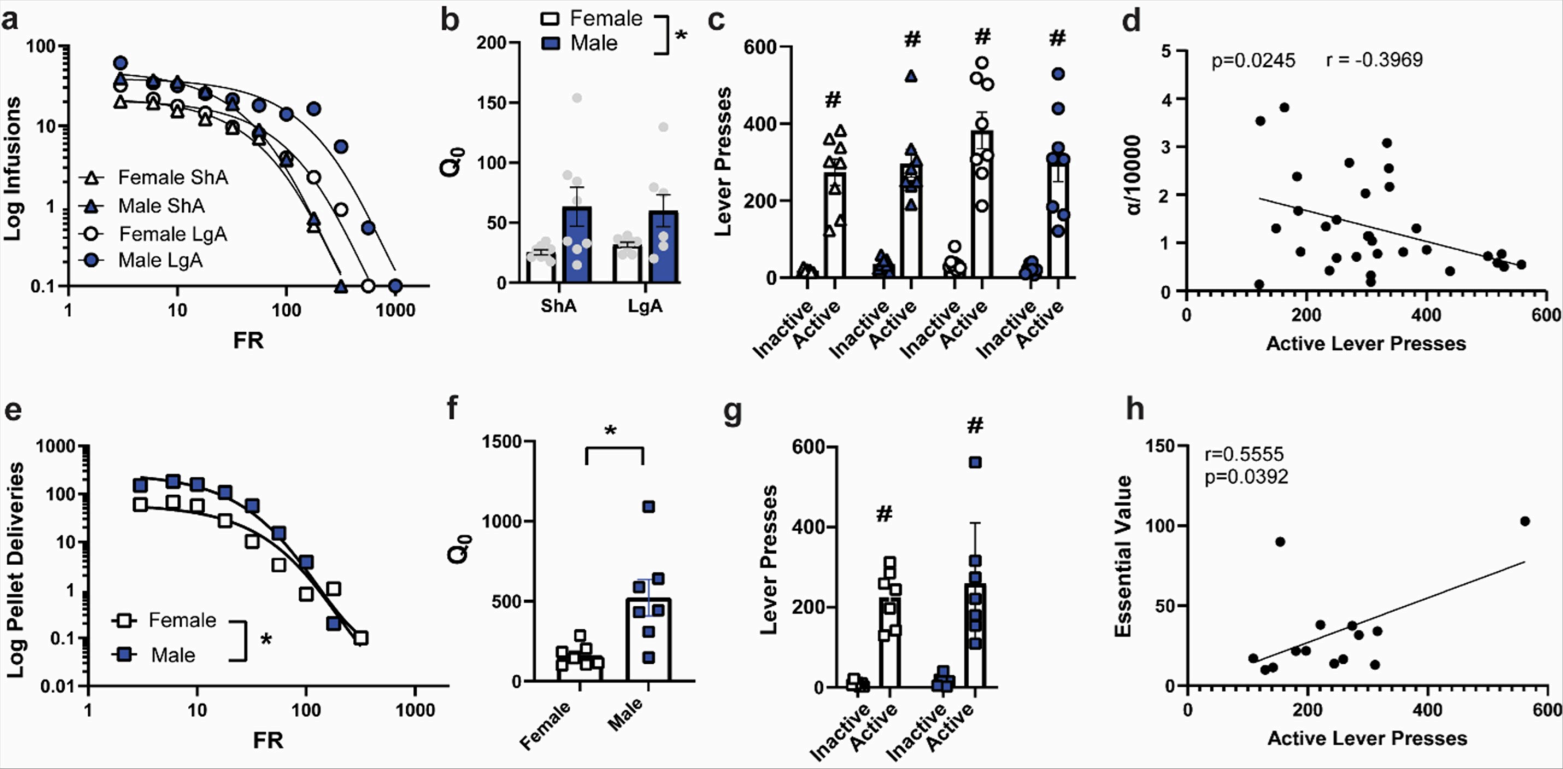
Economic demand for intravenous oxycodone and food pellets differed by sex and was related to cue-primed relapse. a. Demand elasticity (α) for intravenous oxycodone did not differ between access conditions. b. Q_0_ was greater in male than female oxycodone rats, independent of access condition. c. There were no effects of sex or access condition on lever pressing during a cue-primed relapse test; all groups pressed the active lever more than the inactive lever. d. Alpha was inversely correlated with active lever pressing during the relapse test such that less elastic demand for oxycodone was associated with greater cued seeking. e. Male rats displayed less elastic demand for food pellets. f. Q_0_ was greater in male than female food rats. g. There were no sex differences in cued relapse to food seeking; both sexes pressed the active lever more than the inactive. h. Essential Value of food was positively correlated with active lever presses during the relapse test. * = p<0.05 comparing males to females. # = p<0.05 comparing Active to Inactive lever. All panels depict mean ± SEM.

Demand elasticity, or alpha (α), for intravenous oxycodone did not differ between access conditions (Fig. 2a). Q_0_ was greater in male than female oxycodone rats, independent of access condition [main effect of Sex: F_(1, 28)_ = 9.708, p > 0.0042; Fig. 2b]. There were no effects of Access or Sex on P_max_ or Essential Value (not shown). There were no effects of Access or Sex on lever pressing during a cue-primed relapse test; all groups pressed the active more than the inactive lever [main effect of Lever: F_(1,56)_ = 179.4, p < 0.0001; Fig. 2c]. Alpha was inversely correlated with active lever pressing during the relapse test such that less elastic demand for oxycodone was associated with greater number of active lever presses during the cued relapse test (Fig. 2d).

Male rats displayed less elastic demand for food pellets than female rats [t_(12)_ = 3.521, p < 0.01; Fig. 2e]. Q_0_ was greater in male than female food rats [t_(12)_ = 3.093, p > 0.01; Fig. 2f]. There were no sex differences in cued relapse to food seeking; both sexes pressed the active lever more than the inactive [main effect of Lever: F_(1,12)_ = 52.78, p < 0.0001; Fig. 2g]. There were no effects of Sex on P_max_ or Essential Value, but Essential Value of food was positively correlated with active lever presses during the relapse test (Fig. 2h).

### Novel Object Recognition (NOR)

**Figure 3:**
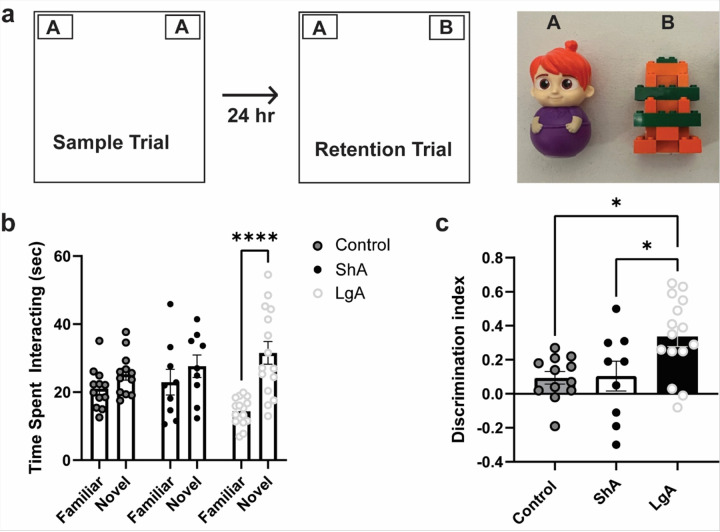
Recognition of the novel object was enhanced in rats that had long-access to oxycodone self-administration. a. Schematic of the novel object set-up. Two objects with distinct visual and tactile properties were used (Objects “A” and “B”, far right). On the Sample day, two identical objects were placed in the arena. During the Retention trial 24 hr later, one familiar object from the prior day was placed in the arena along with a novel object. b. The amount of time spent with the objects during the Retention trial differed by group, with only the LgA group spending more time with the novel object than the familiar. c. The discrimination ratio differed by group (Time spent with novel object – Time spent with familiar object/total time spent with both objects), with the LgA group displaying greater discrimination ratio than the ShA and Control groups. **** = p<0.0001 comparing familiar to novel; * = p<0.05 for respective comparisons. Data is depicted as mean ± SEM.

Data from the NOR were not analyzed with Sex as a factor due to low n/sex after excluding rats that escaped the chamber or knocked over the objects. When time spent interacting with the novel and familiar objects were compared between groups, there was a significant Group × Object interaction [F_(2, 66)_ = 4.524, p = 0.0144; Fig. 3b]. Post-hoc tests found that only the LgA condition spent more time with the novel object than the familiar object (p < 0.0001). There was a significant main effect of Group for the Discrimination Index [F_(2, 33)_ = 5.598, p = 0.0081; Fig. 3c], with post-hoc tests finding that the LgA group showed better discrimination for the novel object than the other groups (p’s < 0.05). Multiple linear regression found that the variance in the discrimination index was not explained by the oxycodone intake/seeking factors included in the model.

### Probabilistic Reversal Learning (PRL)

There was no Group × Sex × Day interaction for the number of trials completed across the 15 days of the PRL task, but there was a significant effect of Sex [F_(1,40)_ = 46.898, p < 0.0001]. To follow up on the main effect of Sex, a 2-way Sex × Day ANOVA was conducted on the number of trials completed by Control rats only, again finding a main effect of Sex, [F_(1,12)_ = 19.86, p < 0.001], with male rats engaging in approximately twice as many trials as females throughout the 15 sessions. Thus, the effects of oxycodone access on PRL were analyzed separately for males and females.

**Figure 4:**
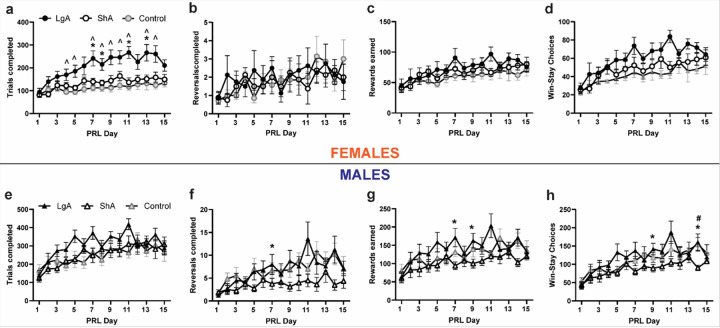
Probabilistic reversal learning was not globally impaired by a history of oxycodone. a. Female LgA rats completed more trials than ShA and Control rats. b. There were no group differences in the number of reversals completed, with reversals increasing over time. d. The number of rewards earned also increased over time, independent of group. d. There were no group differences in the number of win-stay choices between female groups, with all groups showing increases over days. e. Male rats completed an increasing number of trials across the 15 days with no group differences. f. There was a Group × Day interaction for the number of reversals completed with post-hoc tests failing to identify group differences on any day. g. There was a Group × Day interaction for the number of rewards earned, with LgA showing increased rewards on two days of PRL. h. There was also a Group × Day interaction for the number of win-stay choices, with LgA rats engaging in more win-stay behavior on two days. * = p<0.05 LgA vs. ShA; ^ = p<0.05 LgA vs. Control; # = p<0.05 ShA vs. Control. All panels depict mean ± SEM.

For female rats, there was a Group × Day interaction for total PRL trials completed [F_(7.630, 76.30)_ = 2.101, p = 0.0484], with LgA rats completing more trials than both Control and ShA on several days (Fig. 4a). There was only a main effect of Day for the number of reversals completed [F_(5.154, 103.1)_ = 2.891, p = 0.0165; Fig. 4b], with no Group differences. The same was observed for the number of rewards earned [F_(5.574, 111.5)_ = 11.43, p < 0.0001; Fig. 4c], the number of win-stay choices [F_(4.680, 93.59)_ = 13.00, p < 0.0001; Fig. 4d], and lose-shift choices [F_(6.992, 139.8)_ = 3.191, p = 0.0037; not shown], with all groups showing increases over the course of the 15 days. Because LgA females completed more trials than other groups throughout the 15-day period, we also computed the same analyses accounting for the number of trials completed, finding no effects for reversals (Suppl. Fig. S2a) or rewards (Suppl. Fig. S2b). There was a main effect of Day for the number of win-stay choices/trial [F_(6.319, 126.4)_ = 3.340, p = 0.0037; Suppl. Fig. S2c]. There were no significant differences in the number of lose-shift choices per trial (Suppl. Fig. S2d).

Male rats completed an increasing number of trials across the 15 days [F_(3.963, 79.25)_ = 11.86, p < 0.0001; Fig. 4e] with no group differences. There was a Group × Day interaction for the number of reversals completed [F_(11.48, 114.8)_ = 2.214, p = 0.0167; Fig. 4f]. Post-hoc tests found no group differences in reversals on any day of PRL. There was a Group × Day interaction for the number of rewards earned [F_(12.34, 123.4)_ = 2.222, p = 0.0135; Fig. 4g], with LgA showing increased rewards on one day of PRL. There was also a Group × Day interaction for the number of win-stay choices [F_(14.20, 142.0)_ = 1.855, p = 0.0355; Fig. 4h], with LgA rats engaging in more win-stay behavior on two days. While male rats did not differ in the number of trials completed, we computed the same analyses accounting for the number of trials completed. The number of reversals completed per 100 trials differed by Day [F_(4.529, 90.57)_ = 5.667, p = 0.0002; Suppl. Fig. S2e]. There were no effects of Group or Day on the number of rewards earned per 100 trials (Suppl. Fig. S2f). Win-stay choices per trial differed by Day [F_(4.526, 90.52)_ = 6.418, p < 0.001; Suppl. Fig. S2g] but not Group. There was a significant Group × Day interaction for the number of lose-shift choices per trial [F_(9.587, 57.52)_ = 2.703, p = 0.0095; Suppl. Fig. S2h]. Post-hoc tests found no significant differences between groups on any day.

The multiple linear regression for PRL data found that 45% of the variance in the number of reversals/100 trials was explained by the model (F_(6, 25)_ = 3.436, p = 0.0130). The number of active lever presses during the relapse test (β = 0.004570, p = 0.0422) and sex (β = 1.346, p = 0.0028) were significant predictors of this measure. Only in males were increased active lever presses during the relapse test associated with greater reversals/100 trials.

Sex × Group ANOVAs were used to assess PRL performance solely on the day of perfusion when the 90-min trial would influence c-fos expression. When analyzing behavior (reversals, rewards, win-stay, and lose-shift) per trial, there were no Sex × Group differences for any variable. However, the total number of trials completed did differ by Sex [F_(1, 39)_ = 18.19, p < 0.001], potentially explaining significant effects of Sex on the number of reversals completed [F(1, 39) = 9.303, p = 0.0041] and rewards earned [F(1, 39) = 19.21, p < 0.0001], with males completing more trials, reversals and earning more rewards. Thus, correlations were conducted between c-fos expression and specific behaviors (e.g. reversals) per trial completed (see below).

#### C-fos expression

One female ShA rat had poorly perfused tissue and was unable to be used for immunohistochemistry, leaving n = 7 for this group. Individual c-fos expression values were excluded from analysis that were more than two standard deviations above (VO: 1 female Control; aLO 1 female LgA; MO: 1 male Control) or below (PL: 1 male ShA rat; Cg1: 1 female rat; VO: 1 male ShA and 1 female LgA) the mean.

**Figure 5:**
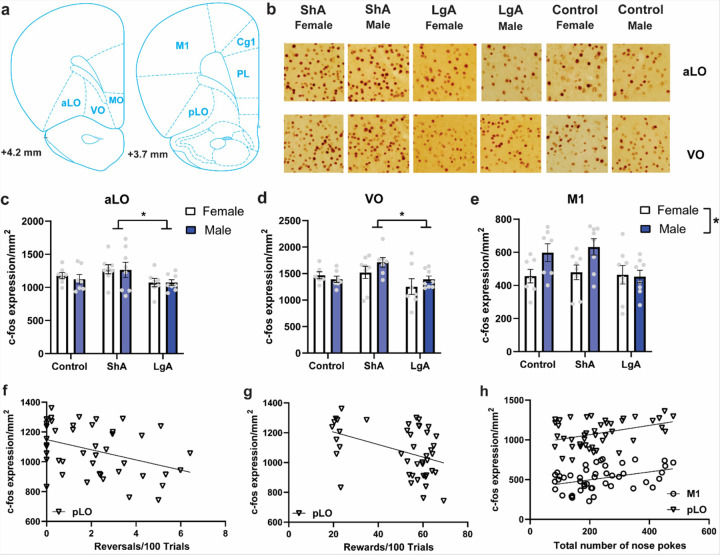
Cortical c-fos expression following the final PRL session was affected by both sex and oxycodone access. a. Two images were acquired from labeled cortical regions for each rat and c-fos/mm^2^ quantified. b. Representative images from the regions showing increased c-fos expression in the ShA condition. c. In the aLO, there was a main effect of Group [F_(2, 38)_ = 3.570, p=0.038], but no effect of Sex and no interaction. The ShA group had increased expression relative to the LgA group. d. A similar pattern was found for the VO. e. In the M1 cortex, a significant effect of Sex was observed, with male rats having greater expression than females. f. C-fos expression in the pLO was negatively correlated with both the number of reversals/100 trials [r_(44)_ = −0.384, p=0.0099] and (g) the number of rewards/100 trials on the last day of the PRL task [r_(44)_ = −0.4178, p = 0.0048]. h. M1 c-fos expression was positively correlated with the total number of nose pokes emitted [r_(45)_= 0.3821, p=0.0096], as was pLO c-fos expression [r_(44)_= 0.3753, p=0.0121]. Data in Panels C-E is depicted as mean ± SEM.

In the PL, Cg1, MO and pLO cortices, there were no effects of Group, Sex or Group × Sex interactions for c-fos expression (Suppl. Fig. S3). In the aLO, there was a main effect of Group [F_(2, 38)_ = 3.570, p = 0.038], but no effect of Sex and no interaction (Fig. 5c). A post-hoc test comparing groups, independent of Sex, found that the ShA group had increased expression relative to the LgA group. A similar pattern was found for the VO [F_(2, 36)_ = 4.005, p = 0.0269; Fig. 5d]. In the M1 cortex, a significant effect of Sex was observed [F_(1, 39)_ = 5.945, p = 0.0161; Fig. 5e], with male rats having greater c-fos expression than females.

Pearson’s correlations were conducted between c-fos expression and behavior during the PRL task on the day of perfusion. C-fos expression in the pLO was negatively correlated with both the number of reversals/100 trials [r_(44)_ = −0.384, p = 0.0099; Fig. 5f] and the number of rewards/100 trials [r_(44)_ = −0.4178, p = 0.0048; Fig. 5g]. Expression in M1 was positively correlated with the total number of nose pokes emitted [r_(45)_ = 0.3821, p = 0.0096; Fig. 5h], as was c-fos expression in the pLO [r_(44)_ = 0.3753, p = 0.0121; Fig. 5h].

## Discussion

In the present study, long-access to oxycodone self-administration yielded increases in oxycodone intake with no effect on economic demand for oxycodone, indicating that elasticity of demand is a separate construct than intake. In agreement with our prior study (Wilkinson et al. 2023), demand elasticity for intravenous oxycodone was negatively correlated with oxycodone seeking during a cue-primed relapse test, with reduced elasticity (reduced willingness to decrease intake when “cost” for drug increases) being associated with increased cued seeking. In rats that had self-administered food, a similar effect was found with the essential value of food positively correlated with active lever presses during a relapse test. Thus, in this model of economic demand, which entails increasing the effort needed to earn a reinforcer over successive days, elasticity of demand is related to cued seeking after abstinence, potentially relying on the same neurocircuitry. Oxycodone self-administration did not lead to impairments in either recognition memory or cognitive flexibility. In fact, long-access to oxycodone was associated with better recognition memory, or at least a preference for a novel object. Economic demand was not predictive of cognitive flexibility, but drug seeking during a cued relapse test was in a sex-dependent manner. Finally, PRL performance was related to cellular activity in the OFC. While exhibiting similar PRL behavior on the day of perfusion, the ShA group displayed greater c-fos expression in two OFC subregions, indicating that greater cellular activity was necessary to maintain similar performance.

### Relationship between intake and economic demand.

Despite increased total oxycodone intake during self-administration training, LgA rats did not display reduced elasticity of demand for oxycodone relative to ShA rats. This indicates that oxycodone exposure is not a predictor of persistent drug seeking when the cost of oxycodone increases. In contrast, providing male rats LgA (12 hr/day) to fentanyl vapor self-administration increases Essential Value and P_max_ for fentanyl relative to a ShA condition (1 hr/day) (McConnell et al. 2021). In male and female rats, intravenous morphine intake during training is associated with reduced elasticity of demand during economic demand analyses (Harris et al. 2024). Here, males had higher Q_0_ for both food and oxycodone. No sex differences in Q_0_ are observed in demand for morphine (Harris et al., 2024), indicating that oxycodone may be different from other opioids in this regard. Alternatively, had we compared 12 hr to 1 hr daily access to oxycodone here, we may have observed differences in demand elasticity.

#### Recognition Memory

No deficits in recognition memory were observed in oxycodone-experienced rats. In fact, LgA rats spent more time with the novel object. The rodent literature is mixed with regards to the effects of non-contingent heroin and oxycodone on object recognition, with some finding increases and some decreases and no clear effect of abstinence on this behavior (Zhong et al. 2018; Francis et al. 2022; Wan et al. 2023). Thus, one interpretation of the present data is that abstinence from long-access oxycodone increases recognition memory. Alternatively, the preference for novelty may be increased. In men that were in acute abstinence from heroin or maintained on methadone therapy, no deficits in spatial recognition or episodic memory were observed (Baldacchino et al. 2019). This is in contrast to findings that ongoing heroin use, ongoing non-prescription opioid use, as well as brief (3 weeks) and protracted (1 year) abstinence from heroin is accompanied by deficits in episodic memory (Ersche et al. 2006; Fishbein et al. 2007; Kroll et al. 2018). Thus, the human literature largely supports the presence of deficits in episodic memory during ongoing opioid use and following abstinence with some evidence of the same following non-contingent, but not contingent, opioid use in rodents.

#### Cognitive Flexibility

While the data from the PRL task did not find overt deficits in reversal learning in either oxycodone group here, we did find more subtle differences in decision making akin to those found in humans. When no differences in performance were found in a probabilistic reward-punishment task in persons with OUD currently on replacement therapy relative to healthy controls, the two groups employed different strategies in the task. The OUD group showed increased lose-shift behavior relative to healthy controls (Myers et al. 2016), indicating that the OUD group altered their response strategy following a negative outcome, while controls were more likely to maintain a consistent response strategy that resulted in long-term, rather than short-term, wins. However, another study found the opposite – that persons in protracted abstinence from heroin use (minimum of 3 months of abstinence and average abstinence length of 2.9 years) have reduced aversion to loss compared to healthy controls (Ahn et al. 2014). Taken together, ongoing opioid agonist intake may lead to increased lose-shift behavior while abstinence produces the opposite. Only males showed group differences in win-stay/lose-shift behavior in the present study, with behavior approximating that of abstinent human heroin users (less lose-shift behavior than controls). When a similar PRL task was used to test male rats abstinent from fentanyl IVSA for at least three weeks, the fentanyl group performed more reversals than the sucrose self-administering control group, with almost no lose-shift behavior in either group (Wheeler et al. 2023). In a different measure of cognitive flexibility – an operant attentional set-shift task – male and female mice had deficits specifically with the extradimensional shift aspect of the task following two weeks of abstinence from remifentanil, but not with other aspects of the task (Anderson et al. 2021). Thus, as in humans, abstinence from self-administration of opioids in rodents may be associated with more subtle changes in some, but not all, aspects of cognitive flexibility.

Abstinence from opioids may also result in recovery of executive function, at least in some domains (Pau et al. 2002; Rapeli et al. 2006). Deficits in reversal learning and recognition memory in opioid users are observed in participants with ongoing use (Kroll et al. 2018) or maintenance on opioid replacement therapies such as methadone or buprenorphine (Myers et al. 2016; Tolomeo et al. 2019; Regier et al. 2024). In fact, opioid users on methadone maintenance exhibit poorer reversal learning than abstinent heroin users (Verdejo et al. 2005). Opioid replacement therapies impair other cognitive domains as well. Compared to abstinent heroin abusers, patients on methadone maintenance demonstrate lower working memory accuracy and perform slower on visuo-spatial attention, processing speed, and cognitive flexibility tasks (Verdejo et al. 2005). Thus, had we examined cognition during ongoing self-administration, we may have observed effects on cognition. To our knowledge, no rodent study has examined the effects of ongoing self-administration on any measure of cognition, an important future direction for such work.

Another consideration is that the use of additional drugs (polysubstance use; PSU) is not often considered in human studies. Those that have explicitly assessed opioid-alcohol PSU have found worsened deficits in set-shifting and cognitive flexibility in participants reporting use of both opioids and alcohol relative to those using opioids only both during ongoing use and after 3 weeks of abstinence (Fishbein et al. 2007; Arias et al. 2016). Several publications on the topic either do not assess alcohol use or do not consider it in data analysis. It is possible that opioid PSU produces cognitive deficits while opioid-only use does not or produces deficits that recover with time.

The number of PRL trials completed was greater in female LgA rats relative to other groups. This was not due to a greater number of lever presses during self-administration training, as Controls exhibited the most lever presses during self-administration training. This was also not likely due to acute locomotor effects of oxycodone, as the PRL task occurred more than 15 days after the last self-administration session. Finally, the regression model found that both sex and active lever pressing during the cued relapse test were predictors of the number of reversals, with greater seeking accompanied by a greater number of reversals in male rats.

#### C-fos expression

C-fos expression in the anterior and ventral OFC subregions was greater in the ShA group relative to the LgA and control groups despite similar PRL performance on the day of perfusion. In PFC regions including the OFC, PL and Cg1, c-fos expression reflects strong excitatory glutamatergic drive and modulatory dopaminergic input during behaviorally relevant stimuli or drug exposure (Merchant et al. 1996; Herry and Mons 2004; Siahposht-Khachaki et al. 2018). Thus, the present results indicate that greater cellular activity and/or glutamate and dopamine release was necessary to maintain similar performance in ShA rats. A similar result was found in human opioid users, wherein the absence of deficits in risky decision-making relative to healthy controls, increased OFC activity during the task was observed (Ersche et al. 2005a).

## Conclusion

Extended access to oxycodone self-administration does not lead to increased seeking under high effort conditions or after a period of abstinence, indicating that the motivation to seek oxycodone is not due to the amount of drug exposure. Oxycodone intake and seeking are not associated with global deficits in recognition memory or reversal learning. Instead, rats demonstrate a subtle change in lose-shift behavior, consistent with data from abstinent opioid users. Future work will examine whether impaired memory or reversal learning/decision-making is observed during ongoing opioid self-administration in rats.

## Supplementary Material

This is a list of supplementary files associated with this preprint. Click to download.

- SupplementalInformation.docx
